# Improving responses to safety incidents: we need to talk about justice

**DOI:** 10.1136/bmjqs-2021-014333

**Published:** 2022-01-20

**Authors:** Alan Cribb, Jane K. O'Hara, Justin Waring

**Affiliations:** 1 Centre for Public Policy Research, King's College London, London SE1 9NH, UK; 2 School of Healthcare, University of Leeds, Leeds, UK; 3 Yorkshire Quality & Safety Research Group, Bradford Institute for Health Research, Bradford, UK; 4 Health Services Management Centre, University of Birmingham, Birmingham, UK

**Keywords:** safety culture, patient safety, healthcare quality improvement, health policy

The promotion of a ‘just culture’ features prominently in patient safety research and policy. This has come about, in part, from a recognition that a so-called ‘blame culture’ discourages openness and learning.^1^ It also reflects a growing understanding that people caught up in safety events (patients, their families and healthcare staff) can experience feelings of sadness, guilt and anger, and need to be treated fairly and sensitively. Despite this growing understanding, there remain significant difficulties in listening to and involving patients and families in the organisational responses to safety incidents,^2–4^ and for healthcare staff, a blame culture often persists.^5^ These, in turn, lead to a sense of sustained unfairness, unresponsiveness and secondary harm. Yet, despite aspirations for a ‘just culture’, the idea of justice itself is rarely discussed explicitly or in depth. We contend that part of the ongoing muddle about safety cultures stems from this lack of focused attention on the nature and implications of justice in the field of patient safety. Put simply, we need to talk about justice.

There is, however, no simple, agreed and all-encompassing definition of justice, except perhaps at a very abstract level where justice means ‘everyone giving and getting what they owe and are owed’. But hiding underneath this large umbrella there are different conceptions of justice which tell rather different stories about what kinds of things are owed, and by and to whom. If those involved in patient safety are to respond respectfully and wisely to concerns about justice then an important first step is to attend more directly to questions about the nature of justice.

In what follows, we summarise three accounts of justice with relevance for patient safety, highlight some of the dilemmas and uncertainties that arise from these competing conceptions and briefly indicate how the field of patient safety might begin to address these complexities.

## Conceptions of justice and patient safety

To help open up the value questions behind safety policies we first summarise some of the relevant conceptions of justice:

*Justice as facing sanctions*. A commonplace conception of justice that is widely accepted in relation to punishment for crimes is ‘*retributive justice’*. This is a ‘corrective’ approach to justice—whereby an offender is expected to pay some penalty for the harm inflicted on others or done to the social fabric. Although punishment can be directed towards other purposes—such as deterrence or reform—punishment is warranted when it is specifically applied to only those people who have committed wrongs. Retributive justice is usually distinguished from revenge because the former is measured and insulated from personal emotions.^6^ This idea connects with a broader principle of ‘justice as desert’—that people can deserve to be given praise or blame, sometimes including rewards or sanctions, so as to reflect some combination of the conscientiousness and success of their efforts.

*Justice as no blame or qualified blame*. There is a concern that a blaming and sometimes ‘punitive’ mindset can be applied too widely and that this in itself is unjust. In key respects, this outlook is compatible with the retributive principle—which holds that only people who deserve penalties should face penalties—but it is often interpreted as being antiretributive. It combines two different strands of thought. The first strand completely shifts focus away from attaching blame towards a focus on understanding and tackling the contributory or system factors that produce failures, that is, it ‘suspends’ desert. The second strand, by contrast, ‘extends’ desert, that is, it sees accountability as extending beyond the person at the ‘sharp-end’ to the many people who have collective responsibility for the organisation and performance of systems. Both of these strands are usually justified in ‘*utilitarian’ terms*; that is, by reference to the aggregate benefit they can produce in system learning and ultimately harm prevention.^7^ The second strand can also be seen as an instance of ‘*distributive justice*’ which is about the fair distribution of benefits and burdens in society^8^—in this case ‘dividing up’ responsibility fairly within organisations.

*Justice as repair*. Another conception takes a ‘*restorative*’ view of justice. Like retributive justice, this is a ‘corrective’ conception, that is, it is about seeking to ‘make things right’. The core concern is how to repair the damage done to those affected by failings, including damage to the social and moral fabric, such as to trust and relationships.^9^ What matters here is that some genuine effort is made to ‘mend’ the damage experienced by victims. As with retributive justice, the aim need not be a utilitarian one—seeking to ‘make right’ may or may not produce the most benefit overall and that is not its point.

These three conceptions have been highlighted because they are evident in changing patient safety discourses. Although only a limited number of healthcare harms fall into the category of crimes, it has been commonplace in the health and care workplace for people to be censured or face sanctions when deemed responsible for certain failures, especially those that risk or cause harm to others. In other words, something like retributive justice underlies the ‘blame culture’ that is said to inhibit learning and improvement in health and care services.

By contrast, the promotion of a ‘just culture’ reflects the recognition that patient harm is rarely the sole consequence of individual wrongdoing, but rather is made more likely by latent factors located in the organisation of care.^10 11^ This system approach—on which much patient safety policy is built—replaces ‘fault finding’ and ‘blame’ with ‘learning’ and ‘risk reduction’. This broadly leads towards a ‘no blame’ conception, although some proponents of a ‘just culture’ characterise their approach in terms closer to a ‘qualified blame’ conception, where blame is not completely eliminated, but rather is dispersed or shared widely within organisations. In addition, some advocates of a ‘just culture’ also promote a restorative view, arguing that this involves organisations responding to the needs of those who have been harmed—first and foremost patients and families, but also professionals who are sometimes seen as ‘second victims’ of safety failings (eg, when they just happen to be on duty at the time a safety flaw became manifest).^5^ To be *just* in this sense will include such things as honestly sharing accounts of what happened and being supportive to patients and implicated staff so they do not feel abandoned. It may also include apologies, agreement on remedial action and compensation.^12^

## Dilemmas and uncertainties for patient safety policy and practice

These multiple understandings of justice create some important challenges for patient safety both in principle and in practice. The core question for all those interested in designing and implementing more ‘just’ safety policies and procedures is working out which conceptions of justice they should work with and emphasise. We cannot just ‘pick’ one conception of justice and neglect the others because they each make a relevant claim on us. The values they highlight—*proportionate sanctions*, *shared learning and accountability* and *repairing damage—*all seem important. One superficially plausible response is simply to say that we should address them all simultaneously. Indeed, on the surface, these conceptions are not inherently incompatible. But there are tensions as well as compatibilities between the conceptions, and potential combinations can have very different emphases. This raises important ethical questions about better and worse combinations that we are arguing need more explicit attention.

But these are by no means questions purely for philosophers or theorists; rather they are questions for everyone interested in dealing with patient safety in an ethical and just way. The existence of different understandings of justice gives rise to important dilemmas and uncertainties about what to do in practice. Indeed, if anything, these challenges are compounded in practice. For example, different stakeholders within the healthcare system may stress different interpretations of justice. Further, there may often be a gap between an organisational narrative about justice, and the experience and perceptions of those affected by safety events when conceptions of justice suddenly come into sharp relief. These complications can be indicated by some illustrative scenarios:

- An organisation may officially advocate ‘no blame’ or talk about such things as ‘corporate manslaughter’ regulations that push accountability higher up the system, yet in day-to-day practices staff may still feel they are being treated retributively.
- An organisation officially accepts system responsibility for patient harm, but the patient might perceive this as a way for any and all individuals (including senior managers) to evade warranted sanctions.
- Both professionals and patients may worry about how meaningful or helpful some forms of ‘moral repair’ are if and when they do not seem to be combined with a genuine acknowledgement of fault on anybody’s part.

## What should be done?

This essay is primarily about identifying and ‘naming’ this issue as a critical first step towards more extensive and open debate. However, to conclude, we suggest some implications for those working in patient safety scholarship, policy and practice.

### Extending scholarship

The scholarship on ‘just cultures’ already includes some insightful conceptual work on justice in safety. However, work to date has not directly tackled the philosophical and ethical questions entailed by balancing different conceptions of justice together. For example, Weiner *et al* looked at justice in relation to safety incident reporting and helpfully set out how various dimensions of justice might be incorporated within a just culture.^13^ Their overall orientation—familiar across the ‘just culture’ literature—is that there is a need to move away from a punitive culture. This is reinforced by more recent work on the harmful effects on professionals and organisations of retaining ‘retributive justice mechanisms’—such as disciplinary action—within a just culture framework.^14^

This broad direction of travel is widely accepted, but it leaves unanswered the key ethical question of what, if any, kinds and levels of sanctions should remain. Important arguments have been advanced by scholars sympathetic to a systems approach to safety that this should not mean that individual accountability falls away completely.^15^ Similarly, advocates of restorative justice have suggested that ‘unjust absolution’ (and not only ‘unjust retribution’) is a block to moral repair.^12^ In other words, they invite us to consider when the absence of blame may be unjust. It is arguable that the only way to completely avoid some retributive element is to adopt a wholesale ‘no blame’ approach. This might seem to be a credible pragmatic position, and one that can be defended on utilitarian terms, but it will not be accepted as an account of justice from many perspectives. By definition, safety policies are geared towards harm prevention, and will have a utilitarian emphasis. However, that does take the ethical debate very far. Even sticking to a utilitarian lens there are a broad range of—sometimes incommensurable—potential harms to be considered, compared and somehow ‘weighed’. This includes, for example, the potential ‘dignitary harms’ or even ‘moral harms’ produced both by safety incidents and our responses to them.^16^ Scholarly work is needed on these themes.

### Extending debate

More urgently, these questions need to be tackled at a practical level within the patient safety community, by practitioners and by patient advocacy groups. Patient safety leaders should begin thinking about how to facilitate these debates and, we recommend, such debates should:

1. *Avoid assumptions about right answers*.

We should not assume that there is one right answer which can be applied across the board. Different contexts and cases will call for a different balance of values, and these need to be negotiated with input from all stakeholders affected by the policy or practice.

2. *Think about effectiveness but also beyond*.^17^

Empirical evidence about ‘what works’ is an important dimension of thinking about value questions but does not answer them.^18^ It is always a meaningful and important question to ask whether a policy is *just* in addition to asking about its effectiveness.

3. *Make discussions concrete*.

Debates should look at ‘real world’ policies and practices and ask what ideas about justice (in which combinations) they embody. This could, for example, include focusing on realistic scenarios in which stakeholders are invited to work out how best, in concrete terms, to combine emphases on *proportionate sanctions*, *shared learning and accountability* and *repairing damage*. As well as asking about the relative importance of these different values and how best to balance and combine them we need to ask, ‘What would our preferred answers look like on the ground?’

In short, we propose that the ethical balancing acts, involving interpretations of justice, that we have highlighted here need to be deliberated about carefully and openly by all those concerned with advancing the safety of healthcare.
